# Vaterite found in saltwater natural pearls from *Pinctada* Sp. mollusks

**DOI:** 10.1007/s00114-025-02038-3

**Published:** 2025-10-29

**Authors:** Chunhui Zhou, Shiyun Jin, Artitaya Homkrajae, Ravenya Atchalak, Abeer Alalawi

**Affiliations:** 1 Gemological Institute of America (GIA), New York, NY USA; 2 Gemological Institute of America (GIA), Carlsbad, CA USA; 3 Gemological Institute of America (GIA), Bangkok, Thailand

**Keywords:** Marine bivalves, Natural pearls, Biomineralization, Calcium carbonate polymorphs

## Abstract

In this study, we report the detection of vaterite in natural saltwater pearls reportedly found inside *Pinctada* species mollusks, collected from the pearl oyster beds near the water of Kuwait in the Persian (or Arabian) Gulf. This rare polymorph of calcium carbonate was found on both surfaces and/or cross-sectional areas. Raman spectroscopy and X-ray diffraction (XRD) analyses were carried out in order to characterize the structures, which confirmed the presence of vaterite. The results of one pearl are detailed in this article, which showed a predominantly vaterite formation with a calcite central core. Our results showed that vaterite not only existed in freshwater cultured pearls grown in freshwater mussels as previously reported but also can be found in pearls from marine bivalve mollusks. To the best of our knowledge, this is the first time vaterite was conclusively identified in saltwater pearls and pearls of natural origin, and such information provided invaluable insights into biomineralization of calcium carbonate in these unique biogenic gem materials.

## Introduction

Pearls are produced by a variety of mollusk species through biomineralization processes. Unlike the formation of shells as a routine biological function, the formation of pearls is the result of defense mechanism in response to injury or irritation to the mantle tissues. It is a complex combination of biochemical and physiological processes. Majority of saltwater pearls formed by various *Pinctada* species (or “pearl oysters”) contain a nacreous surface, with aragonite being the main mineral structure found in both nacreous and prismatic layers, although calcite formation has also been observed both externally or internally (Perez-Huerta et al. 2014; Manustrong et al. 2019; Sato and Komaru 2019; Surve et al. 2024). Vaterite, the least thermodynamically stable form of calcium carbonate, has only been previously found on the lackluster areas of the surfaces in freshwater cultured pearls, or near the center of freshwater tissue nucleated pearls regardless of their surface luster quality (Qiao et al. 2007; Wehrmeister et al. 2007; Soldati et al. 2008; Ma et al. 2009). Apart from pearls, vaterite as a biomineral, has also been found in gastropod eggshells, the shell of the Antarctic clam *Laternula elliptica*, and fish otoliths (Carlstrom 1963; Hall and Taylor 1971; Gauldie 1986; Nehrke et al. 2012).

Historically, the Persian Gulf, also known as Arabian Gulf, has been considered as the center of the natural pearl trade. The history of natural pearling in Kuwait has played a crucial role in the country’s economy for centuries. Until the early 1930 s, diving for natural pearls was a major industry and the main source of income for Kuwait’s economy (Carter 2005; Al-Maani and Alsharari 2014). Although natural pearling in Kuwait is no longer a prominent industry, its legacy remains a cherished aspect of Kuwaiti cultural heritage.

In this study, we report the detection of vaterite in saltwater natural pearls reportedly sourced from the *Pinctada radiata* at the depths of 6–10 m off the coast of Heir Al-Anan in Kuwait. X-ray diffraction (XRD) data and Raman spectra of one pearl is presented in this short communication as an example.

## Materials and methods

The pearl presented in this article is a white colored, semi-baroque shaped saltwater natural pearl weighing 0.59 carats, reportedly harvested from the *Pinctada radiata* mollusk species, among a group of more than 200 pearls from the same source examined. The pearl has been tested using advanced instruments (X-ray microradiography and energy dispersive X-ray fluorescence) that confirmed its natural and saltwater origin (data not shown).

The X-ray diffraction pattern of the pearl is collected on an Inel Equinox 3000 X-ray diffractometer. The pearl is placed directly onto the sample stage without being crushed into powder. The highest point of the pearl’s curved surface is centered on the sample stage (center of the X-ray beam) and kept at the same height as the top of the sample stage. The pearl is rotated along with the sample stage continuously around the X-ray beam during data collection. The data was collected continuously for over 30 min to ensure that some of the weaker peaks become obvious over the noise.

A Renishaw inVia Reflex micro-Raman spectrometer system with a 50× magnification Leica objective lens and a 514 nm diode laser excitation wavelength at room temperature was used to analyze structural characteristics on various locations of the sample in a scanning range from 100 to 1600 cm^–1^. It is a common technique used to distinguish CaCO_3_ polymorphs (aragonite/calcite/vaterite) with reference to different band positions of carbonate ion (CO_3_^2−^) modes.

## Results

**Fig. 1:**
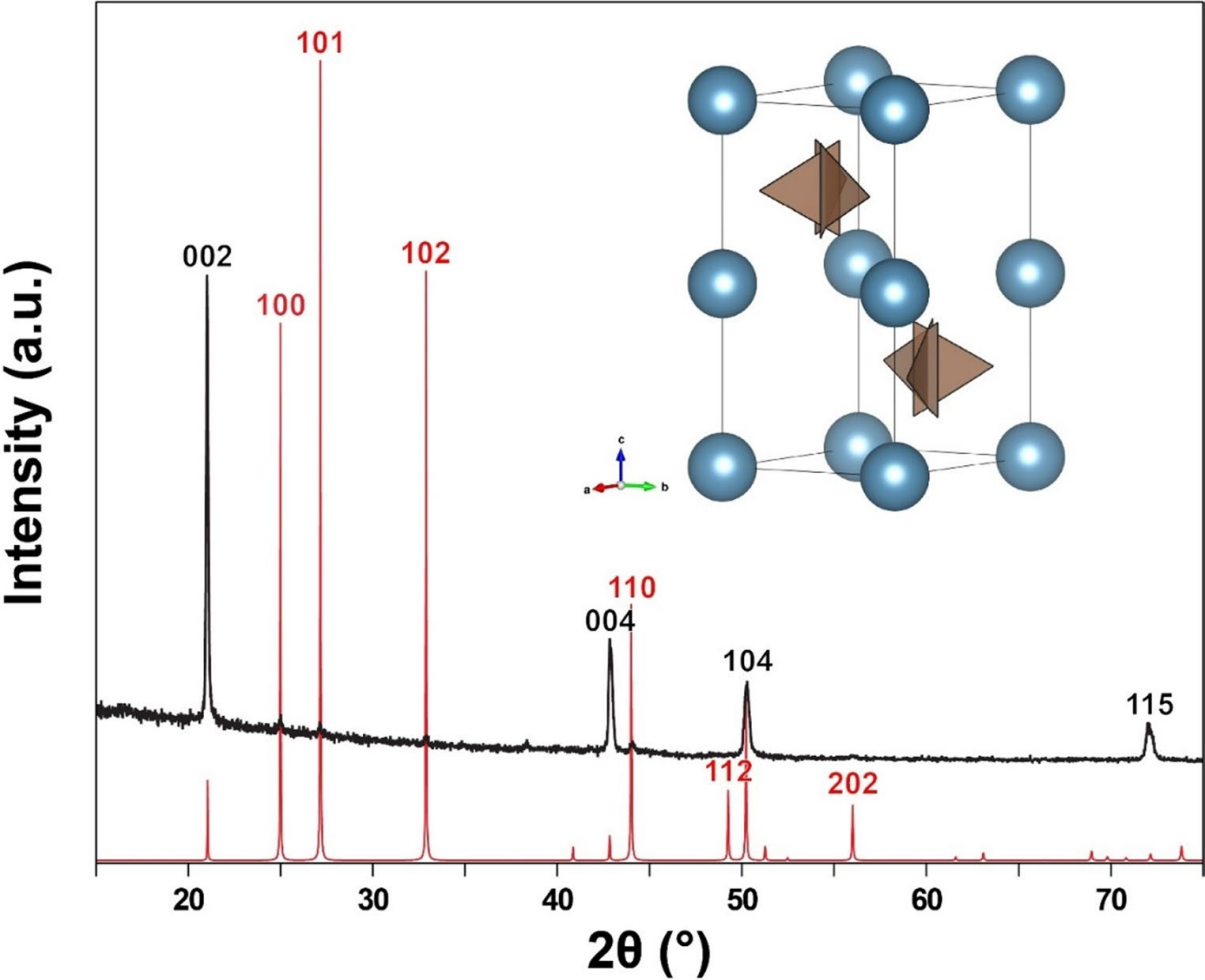
X-ray diffraction pattern collected directly from the surface of the pearl sample. Matching peak positions with the calculated diffraction pattern of vaterite confirms that the only carbonate phase on the surface of the pearl is vaterite. The Miller indices of the strong peaks in the reference pattern are labeled in red, and those of the strong peaks in the collected data are labeled in black. Note that the unmatching peak intensity with the reference pattern is due to the preferred orientations of the vaterite crystals, with c-axis perpendicular to the surface, resulting in disproportionately intense peaks from lattice planes parallel or subparallel to (001). The reference XRD pattern is calculated from the fully disordered hexagonal substructure of vaterite as illustrated above the diffraction pattern

The XRD pattern shows four strong peaks and several very weak peaks. The positions of the peaks match perfectly with the expected peak positions of vaterite, with no indication of any other phase. However, the peak intensities do not match the expected values of vaterite (Fig. 1). This is expected because the vaterite crystals in the pearl are not randomly oriented, which is assumed for the calculated diffraction pattern based on the crystal structure. The strongest peaks expected from vaterite, 101, 102, 100 and 110, are barely noticeable in the collected data, whereas the peaks 002, 004, 104, and 115, which are all from lattice place parallel or subparallel to (001), are disproportionately stronger. This indicates that the vaterite crystals are oriented with the c-axis perpendicular to the surface, same as calcite and aragonite in pearls (Yoshimi et al. 2004; Perez-Huerta et al. 2014).

Vaterite is a metastable polymorph of calcium carbonate with an unusual crystal structure. Unlike the other polymorphs of calcium carbonate, calcite and aragonite, which have the triangular carbonate ions lay flat perpendicular to the c-axis, the carbonate ions in the vaterite are vertically aligned with one of the edges of the triangle parallel to the c-axis. The substructure (basic structure unit) of vaterite is a hexagonal subcell (a = 4.13 Å and c = 8.49 Å) containing two randomly oriented carbonate ions, with a space group symmetry of *P*6_3_/*mmc* (Kamhi 1963). The actual structure of vaterite is much lower in symmetry with larger unit cells due to the ordering of the carbonate ion orientations, and there could be multiple different polytypes (Christy 2017). The reference diffraction pattern in the figure is calculated based on the hexagonal substructure, which is sufficient for confirming the phase as vaterite. The small peak at ~ 38.4° is likely an indication of the actual vaterite structure being lower than the *P*6_3_/*mmc* subcell. Nonetheless, further details of the crystal structure would require higher resolution data collected on pieces isolated from the pearl sample, which is beyond the scope of this study.

**Fig. 2:**
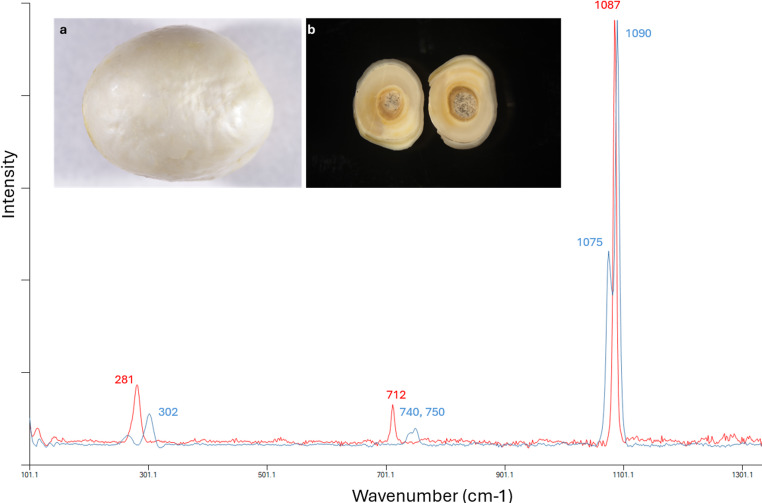
Raman spectra collected on the surface and center region of the cross-section (a and b shown in the inserted images) were concluded to be vaterite (blue) and calcite (red)

The Raman spectroscopy analyses were performed on multiple areas of the sample, including the surface, cross section, and center core. Both surface and outer region of the cross section showed typical Raman shifts for vaterite, with Raman peaks at 740 and 750 cm^−1^ which are assigned to in-plane bending (ʋ4) from internal vibration mode of CO_3_^2−^, and two major peaks at 1075 cm^−1^ and 1090 cm^−1^ that correspond to symmetric stretching (ʋ1) from internal vibration mode of CO_3_^2−^, and series of lattice mode peaks in the low-frequency region with a dominant peak at 302 cm^−1^. In contrast, the center core exhibited corresponding Raman shift peaks at 712 cm^−1^ and 1087 cm^−1^, with dominant lattice mode peak present at 281 cm^−1^, typical for calcite. An example of both Raman spectra from the surface and the center core was shown in Fig. 2, the surface of the pearl looked whiter with frosty and chalky appearance while the center core looked like an aggregation of columnar structures (inserted images in Fig. 2).

## Discussion

The causes and the mechanisms for producing different types of calcium carbonate polymorphs in pearls have not yet been fully understood, some researchers contributed it to the organic matrix which might control the polymorph formation and stabilizes vaterite in pearls (Qiao et al. 2007; Ma and Lee 2006; Wehrmeister et al. 2007). Although the importance of organic components is now widely recognized, their exact function in the formation of pearls and shells has not been deciphered. Other related biomineralization products such as disordered dolomite have been found in a natural saltwater pearl from *Cassis* species (Zhou et al. 2023), signifying the complexity of these biochemical and physiological processes. The frosty and dull appearance of the vaterite surface in the saltwater pearl studied in this project is consistent with the vaterite surfaces in low quality lackluster freshwater pearls previously reported, suggesting a similar mechanism and causation of its formation. The vaterite surface appears to be “nacreous-looking”, showing surface structures similar to the typical stacked aragonite tablets in parallel superimposition (often called the “brick-and-mortar” structure). Environmental changing events such as changes in temperature, viscosity of the extrapallial fluid, diseases or stress may induce vaterite in pearls, similar to vaterite formation in fish otoliths (Gauldie 1993; Soldati et al. 2008). The pearl shown in this article represented a unique and unusual case, which is composed of mostly vaterite, with only the center core made of calcite. In addition, a combination of aragonite and vaterite have also been found in a few saltwater pearl samples in the same studied group (results not shown). This suggested that biomineralization of vaterite in pearls from marine mollusks may occur more frequently than previously thought.

## Conclusion

While many physical and biological factors play important roles in biomineralization of molluscan shells and the reasons for their varying microstructures are not completely clear to us (Checa 2018), biomineralization of pearls has been even less understood, especially for natural pearls. In this study, we demonstrated that vaterite could also be found in pearls originating from marine mollusk species through XRD and Raman spectroscopic analyses. To our best knowledge, this is the first time vaterite has been detected inside of a natural pearl of saltwater origin. It provided a rare opportunity and invaluable insight into our understanding of natural biomineralization process and pearl formation.

## Data Availability

Data is provided within the manuscript.
